# MyD88 in lung resident cells governs airway inflammatory and pulmonary function responses to organic dust treatment

**DOI:** 10.1186/s12931-015-0272-9

**Published:** 2015-09-16

**Authors:** Jill A. Poole, Todd A. Wyatt, Debra J. Romberger, Elizabeth Staab, Samantha Simet, Stephen J. Reynolds, Joseph H. Sisson, Tammy Kielian

**Affiliations:** Pulmonary, Critical Care, Sleep & Allergy Division, Department of Internal Medicine, University of Nebraska Medical Center, 985990 Nebraska Medical Center, Omaha, NE 68198-5990 USA; Department of Environmental, Agricultural & Occupational Health, University of Nebraska Medical Center, 985990 Nebraska Medical Center, Omaha, NE 68198-5990 USA; VA Nebraska-Western Iowa Health Care System, Omaha, NE 68105 USA; High Plains Intermountain Center for Agricultural Health and Safety, Department of Environmental and Radiological Health Sciences, Colorado State University, Ft. Collins, CO USA; Department of Pathology and Microbiology, University of Nebraska Medical Center, 985990 Nebraska Medical Center, Omaha, NE 68198-5990 USA

## Abstract

Inhalation of organic dusts within agriculture environments contributes to the development and/or severity of airway diseases, including asthma and chronic bronchitis. MyD88 KO (knockout) mice are nearly completely protected against the inflammatory and bronchoconstriction effects induced by acute organic dust extract (ODE) treatments. However, the contribution of MyD88 in lung epithelial cell responses remains unclear. In the present study, we first addressed whether ODE-induced changes in epithelial cell responses were MyD88-dependent by quantitating ciliary beat frequency and cell migration following wounding by electric cell-substrate impedance sensing. We demonstrate that the normative ciliary beat slowing response to ODE is delayed in MyD88 KO tracheal epithelial cells as compared to wild type (WT) control. Similarly, the normative ODE-induced slowing of cell migration in response to wound repair was aberrant in MyD88 KO cells. Next, we created MyD88 bone marrow chimera mice to investigate the relative contribution of MyD88-dependent signaling in lung resident (predominately epithelial cells) versus hematopoietic cells. Importantly, we demonstrate that ODE-induced airway hyperresponsiveness is MyD88-dependent in lung resident cells, whereas MyD88 action in hematopoietic cells is mainly responsible for ODE-induced TNF-α release. MyD88 signaling in lung resident and hematopoietic cells are necessary for ODE-induced IL-6 and neutrophil chemoattractant (CXCL1 and CXCL2) release and neutrophil influx. Collectively, these findings underscore an important role for MyD88 in lung resident cells for regulating ciliary motility, wound repair and inflammatory responses to ODE, and moreover, show that airway hyperresponsiveness appears uncoupled from airway inflammatory consequences to organic dust challenge in terms of MyD88 involvement.

**Electronic supplementary material:**

The online version of this article (doi:10.1186/s12931-015-0272-9) contains supplementary material, which is available to authorized users.

## Introduction

Inhalation of organic dusts can result in several inflammatory respiratory diseases, such as asthma, chronic bronchitis, obstructive lung disease, and hypersensitivity pneumonitis, particularly in agriculture-exposed workers [1]. The impact of agriculture exposures in respiratory diseases has recently become a central focus due to its association with increased risk of non-allergic mediated-airway hyper-responsiveness (AHR) and neutrophil-dominant inflammation, whereas providing apparent protection against the development of allergy and eosinophil mediated-asthma [2]. Moreover, currently available therapeutics, including corticosteroids, lack efficacy in the treatment of agriculture-related respiratory diseases [3], underscoring the need to better understand the mechanisms governing the airway inflammatory and pulmonary function response to these environmental exposures.

Organic dusts from agriculture environments are now recognized to be comprised of an abundant and wide diversity of microbial motifs derived from both gram-positive and gram-negative bacteria [1]. Airway inflammatory consequences induced by organic dusts are partially reduced in mice deficient in Toll-like receptor 2 (TLR2) as well as TLR4, but organic dust-induced AHR is not affected by these pathways [4–6]. However, we previously reported a pivotal role for the TLR/IL-1R/IL-18R adaptor protein myeloid differentiation factor 88 (MyD88) that is used by all TLRs (except TLR3) in mediating the lung response to complex organic dusts [4]. Namely, acute organic dust extract (ODE)-induced AHR in conjunction with neutrophil influx and release of inflammatory cytokines/chemokines into the bronchoalveolar space, but not lung parenchyma, was nearly completely abrogated in MyD88 knock-out (KO) mice [4]. Of note, in comparison studies, we did not find a role for IL-1R, and only a limited role for IL-18R, signaling pathway in mediating acute ODE-induced airway inflammatory responses [4]. Based on these findings, we had speculated that MyD88-dependent signaling in lung resident cells, mainly epithelial cells, was mediating airway inflammatory and pulmonary function consequences to acute ODE challenge. However, the importance of MyD88 in epithelial cells and the relative contribution of MyD88 in the lung resident (i.e. epithelial) cell compartment as compared to leukocytes following organic dust exposures remained undefined.

In the present study, we first hypothesized that ODE-mediated decreases in airway epithelial cilia beating and wound repair are mediated by MyD88-dependent signaling. To test this hypothesis, we utilized primary tracheal epithelial cells from MyD88 KO and wild type (WT) animals to compare ciliary motility and cell migration/wound repair responses following ODE treatment. Next, we hypothesized that acute ODE-induced AHR and airway inflammatory consequences were primarily mediated through MyD88-dependent signaling in airway epithelial cells. To test this hypothesis, we investigated respiratory mechanics through invasive pulmonary function testing as well as determined inflammatory cytokine/chemokine production and neutrophil influx following ODE treatment in MyD88 bone marrow chimera mice. The bone marrow chimera approach allowed us to differentiate between MyD88-dependent lung compartment effects, which were broadly defined as lung resident/structural cells versus hematopoietic cells. Our findings highlight novel MyD88-dependent epithelial cell functional responses of ciliary motility and wound repair to organic dust treatments. Furthermore, bone marrow chimeras revealed that AHR to ODE is dependent on MyD88 in lung resident cells, but that inflammatory consequences generally involve both resident and bone marrow derived leukocytes.

## Methods

### Organic dust extract

Aqueous organic dust extract (ODE) was prepared from settled dust collected from horizontal surfaces (~3 feet above floor) of swine confinement feeding operations and extracts were batched prepared utilizing previously described methods [7]. Briefly, dust (1 gm) was placed into sterile Hank’s Balanced Salt Solution (10 ml; Sigma, St. Louis, MO), incubated at room temperature for 1 h, centrifuged for 20 min at 2200 rpm, and the final supernatant was filter sterilized (0.22 μm), a process that also removes coarse particles. Stock ODE was diluted to a final concentration (vol/vol) of 5 % for *in vitro* studies and 12.5 % for *in vivo* studies in sterile phosphate buffered saline (PBS; pH: 7.4; diluent). These respective concentrations have been previously shown to elicit optimal experimental outcomes in airway epithelial cells and mice [5, 8–10] and are well tolerated [10]. ODE (100 %) contains roughly 4 mg/ml of total protein as measured by nanodrop spectrophotometry (NanoDrop Technologies, Wilmington, DE). Endotoxin concentrations are routinely measured throughout experimental studies, and 100 % ODE endotoxin concentrations ranged from 160 to 400 EU/ml as assayed using the limulus amebocyte lysate assay according to manufacturer’s instruction (Sigma). Muramic acid levels, a molecular component of bacterial cell wall peptidoglycans, were determined by mass spectrometry [11] and were 70 ng/mg.

### Animals

All animal procedures were approved by the Institutional Animal Care and Use Committee at the University of Nebraska Medical Center (protocol # 10-054-07) and are in accordance with NIH guidelines for the use of rodents. MyD88 gene knockout (KO) mice on C57BL/6 background expressing the CD45.2 allele were provided by Dr. S. Akira (Osaka, Japan). Age- and sex-matched C57BL/6 mice (The Jackson Laboratory, Bar Harbor, ME) were used as wild-type (WT) controls. For the generation of MyD88 bone marrow chimeras, B6/SJL mice that are congenic for the CD45 allele (CD45.1) on a C57BL/6 background were used as WT animals and were purchased from The Jackson Laboratories. Mice were used between 12 and 16 weeks of age for all organic dust exposure *in vivo* airway studies.

### Exposure murine model

Using our established intranasal (*i.n*.) inhalation exposure animal model [10], mice received treatment with 50 μl of sterile saline (PBS) or 12.5 % ODE under light isoflurane sedation. No mice exhibited respiratory distress throughout the treatment period.

### Ciliary motility assay

Tracheas from unexposed WT and MyD88 KO mice were removed [12], and actively beating ciliated cells from tracheal rings were observed and their motion quantified using Sisson-Ammons video analysis (SAVA; Ammons Engineering, Mt. Morris, MI), a phase-contrast microscopy and computerized frequency spectrum analysis, as previously described [13]. After baseline ciliary beat frequency (CBF) determination, tracheal rings were stimulated with procaterol (100 nM) for 30 min and CBF was again recorded. Rings were also treated with 5 % ODE for up to 24 h with CBF monitoring. During ciliary measurements, tracheal rings were maintained at a constant temperature (24 ± 0.5 °C) by a thermostatically controlled heated stage. Whole-field analysis was performed using software that analyzes the entire captured image of all ciliated cells in a given field. The digital sampling rate was set at 85 frames per second for all experiments. The number of motile cilia was also recorded for each digital video image to rule out selection bias as previously described [14].

### Protein kinase activity

Protein kinase activity was determined in murine tracheal epithelial cells following *in vivo* treatment with intranasal inhalation of saline or 12.5 % ODE at 6 h post-treatment [10]. Epithelial cell lysates were immediately placed in 50 mM Tris-HCl (pH 7.4) lysis buffer with protease inhibitors, and then assayed for protein kinase C (PKCε, PKCα) and protein kinase A (PKA) activity as previously described [8, 10]. Protein kinase activity was expressed in relation to the total amount of cellular protein assayed as picomoles of phosphate incorporated per minutes per milligram (pmol/min/mg).

### Epithelial cell barrier function

Tracheal epithelial cells were harvested from untreated WT and MyD88 KO mice as previously described [15], and were grown to confluence on electric cell substrate impedance sensing (ECIS) 96-well plate arrays (8W1E; Applied Biophysics, Troy, NY) in serum-free medium (1:1: Ham’s F-12 [Hyclone Laboratories, Logan, UT] and DMEM (Gibco, Thermo Scientific, Grand Island, NY). The ECIS system measures transepithelial resistance changes in real-time. Epithelial cell migration following wounding was conducted as previously described [15]. Briefly, an elevated field pulse of 1400 μA at 32,000 Hz was applied for 20 s to epithelial cells that produced a uniform circular lesion of 250 μm in size. Cell migration was tracked over a period of 72 h. Epithelial cell impedance was measured at 4000 Hz, normalized to its value at the initiation of data acquisition, and plotted as a function of time.

### Bone marrow chimera generation

To generate MyD88 bone marrow chimeras, CD45 congenic B6/SJL mice were used that harbor a CD45.1 allele originating from the SJL strain, whereas the remainder of the genome is derived from C57BL/6 mice. These animals represent the WT strain since they express functional MyD88. MyD88 KO mice are on a C57BL/6 background and express the CD45.2 allele, which allows for the discrimination between donor- and recipient-derived leukocytes based on staining with antibodies specific for CD45.1 and CD45.2. The following radiation chimeras were generated in these experiments (donor bone marrow → irradiated recipient): WT → WT, KO → KO, WT → KO and KO → WT. The experimental chimeras were WT → KO and KO → WT, whereas the other two groups (WT → WT and KO → KO) represented controls to rule out any non-specific effects of irradiation on measured responses. Reconstitution of irradiated MyD88 KO animals with bone marrow from WT recipients ensured that leukocytes (i.e. macrophages, neutrophils, lymphocytes) derived from the bone marrow expressed MyD88, whereas lung structural cells (predominately epithelial cells) do not express MyD88 as a result of their radiation resistance [16–18]. The procedure for bone marrow chimera generation was based on our previously published studies [19]. Briefly, bone marrow donor mice were euthanized with sodium pentobarbital and marrow was isolated from the long bones by flushing with sterile 1 × PBS. Recipient mice were placed on antibiotic-supplemented water (1 g/l neomycin and 125 mg/l polymyxin) for 3 days prior to bone marrow transfer and subjected to irradiation (1000 rad) using a RS-2000 Biological System (Rad Source) to destroy the bone marrow. Within 4 to 7 h following irradiation, recipient mice received a retro-orbital dose of 2 × 10^7^ bone marrow cells supplemented with 1 × 10^7^ cells from the spleen to serve as an immediate source of immune cells. Engraftment was allowed to take place over an 8 week period and chimeric animals were maintained on antibiotic-supplemented water for the first 2 weeks to provide protection during transient immunocompromise. At 8 weeks post-transplant, chimeric mice were bled retro-orbitally and cells were stained for flow cytometric analysis using CD45.1 and CD45.2 antibodies (BD Biosciences, Franklin, Lakes, NJ). Only animals that displayed chimerism of greater than 90 % were used in organic dust airway exposure studies.

### Invasive pulmonary function measurements

Total lung resistance (R_L_) and dynamic compliance (Cdyn) was invasively assessed via a tracheostomy tube 3 h following *i.n.* inhalation of saline or ODE treatment as previously described using a computerized small animal ventilator (Finepointe, Data Sciences International, St. Paul, MN) [4, 10]. Dose responsiveness to aerosolized methacholine (0–48 mg/ml) was obtained and reported.

### Bronchoalveolar lavage fluid cell and cytokine/chemokine analysis

In separate animal studies independent of the invasive pulmonary function measurement studies, bronchoalveolar lavage (BAL) was achieved using 3 × 1 ml PBS, and the total cell number recovered from pooled lavages was enumerated and differential cell counts were determined from cytospin-prepared slides (Cytopro Cytocentrifuge, Wescore Inc, Logan, UT) stained with DiffQuick (Siemens, Newark, DE). Cell counts were used to determine the differential ratio of leukocytes with 200 cells per slide per mouse. TNF-α, IL-6, keratinocyte chemoattractant (KC; CXCL1), and macrophage inflammatory protein-2 (MIP-2; CXCL2) were quantitated in the cell-free supernatant of the first lavage by ELISA kits (R&D Systems) with sensitivities of 10.9, 7.8, 15.6, and 7.8 pg/ml, respectively.

### Histopathology

After whole lung lavage, lungs were harvested, inflated with 1 ml of 10 % formalin at a pressure of 20 cm H_2_O for 1 day while submerged in 10 % formalin for optimal preservation of lung parenchymal architecture as previously described [7]. Fixed lung tissues were processed using standard methods, embedded in paraffin, and thin sections (4–5 μM) were stained with hematoxylin and eosin.

### Statistical methods

Data are presented as mean and standard error of mean (SEM). Statistical significance was assessed by one-way analysis of variance (ANOVA) and a two-tailed Mann-Whitney test, where appropriate. For methacholine dose-response curves, two-way ANOVA was applied because two independent variables are involved (i.e. treatment group and methacholine dose), followed by Bonferroni *post hoc* tests when group differences were significant (*P* < 0.05). GraphPad (Version 5.02, La Jolla, CA) software was used. Significance was accepted at *p*-values < 0.05.

## Results

### MyD88 affects the normative ciliary motility slowing response to organic dust

**Fig. 1:**
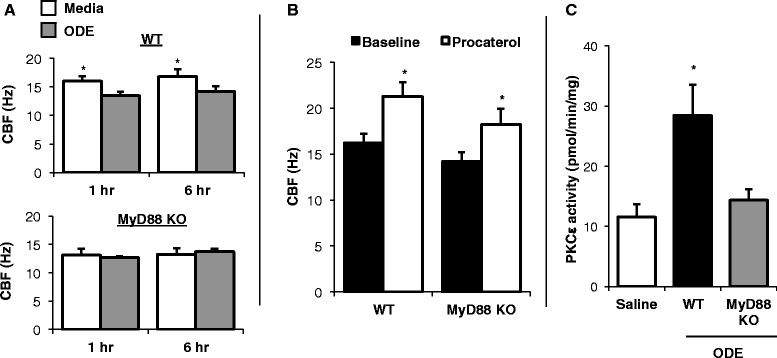
The normative ciliary motility slowing response to ODE is absent in MyD88 KO epithelial cells. WT and MyD88 KO tracheal epithelial cells were investigated for ciliary beat frequency (CBF) by video recording and computer-generated calculations. **a** The normative CBF slowing response to 5 % ODE is lost at 1 and 6 h in MyD88 KO cells. **b** The β_2_ agonist, procaterol, increases CBF in WT and MyD88 KO epithelial cells, demonstrating intact cilia beat response in MyD88 KO cells to non-TLR agonist. Next, at 6 h post-intranasal inhalation of saline or ODE in WT and MyD88 KO mice, mice were euthanized and tracheal epithelial cells were harvested to determine protein kinase C (PKC) epsilon activity. **c** ODE-induced PKCε activity was absent in MyD88 KO epithelial cells. Bar graphs represent mean with SE bars shown of 4–6 independent experiments, each ran in triplicate. Statistical significance denoted by asterisk (*p < 0.05) as compared to media control

The baseline ciliary beat frequency (CBF) in primary tracheal epithelial rings from WT and MyD88 KO mice was 16.23 ± 0.58 Hz and 14.2 ± 1.174, respectively (*n* = 4 independent experiments performed in triplicate; Fig. 1a-b). We have previously demonstrated that ODE slows CBF [14], and these findings were confirmed in WT mice (Fig. 1a). However, the normative ODE-induced CBF slowing response was absent in MyD88 KO epithelial cells at 1 and 6 h post-exposure (*N* = 6 independent experiments performed in triplicate; Fig. 1a). In comparison, a 30 min treatment with the β2 agonist, procaterol (10 nM), increased CBF in both WT and MyD88 KO cells (Fig. 1b), which demonstrates a normative intact cyclic nucleotide-dependent cilia beat response to a non-TLR agonist in MyD88 KO epithelial cells. There was no difference in the number of motile cilia points (an indicator of total number of beating cilia) among groups (data not shown). These studies suggest that MyD88 signaling is important for the effects of ODE on ciliary motility function.

Next, we previously reported that ODE-induced slowing of CBF was dependent on ODE-induced PKCε activity [14], and we also had demonstrated that ODE-induced PKCε activity was absent in ODE-stimulated MyD88 KO lung slices [4]. In proof-of concept studies, WT and MyD88 KO animals were intranasal treated with saline or 12.5 % ODE and at 6 h post-treatment, tracheal epithelial cells were harvested and protein kinase activity was determined. We confirm in primary tracheal epithelial cells that MyD88 signaling is important to ODE-induced PKCε activity because PKCε activation was absent in MyD88 KO, but not WT, tracheal epithelial cells following *in vivo* ODE treatment (Fig. 1c). As a control, ODE inhalation treatment did not significantly alter PKCα or PKA activity at 6 h post-treatment in WT or MyD88 KO animals (data not shown).

### MyD88 KO epithelial cells demonstrate an aberrant wound repair response to ODE

**Fig. 2:**
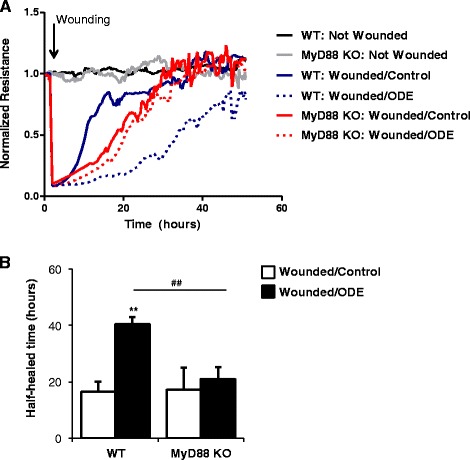
MyD88 KO epithelial cells demonstrate an aberrant wound repair response to ODE. Cell migration (wound repair) response to ODE was assessed in WT and MyD88 KO tracheal epithelial cells by electric cell-substrate impedance sensing (ECIS) system. Cells were treated ± 5 % ODE for 24 h prior to wounding. Cell migration data was continuously collected for up to 72 h post-wounding and displayed as normalized resistance (ohms) (subsequent values divided by initial values). **a** A representative tracing of data from one of 5 independent studies is shown. **b** Bar graph depicts mean (± SEM) time to half-maximal closure in hours (*N* = 5 independent studies). Statistical significance denoted by asterisk (**p < 0.01) as compared to media control and hatchmarks (##*p* < 0.01) denoted by line

Cell migration and wound repair responses were determined using primary tracheal epithelial cells from WT and MyD88 KO mice following pretreatment with or without 5 % ODE for 24 h, whereupon cells were wounded using an automatic electric cell-substrate impedance sensing (ECIS) system. Cell migration data was continuously collected for up to 72 h post-wounding. Consistent with previous studies showing that ODE treatment slows wound closure [20], we demonstrated that ODE dampens cell migration and wound closure in WT epithelial cells (Fig. 2). However, in MyD88 KO cells, cell migration and wound repair responses were not significantly affected by ODE treatment over time (Fig. 2). A representative trace of one of five experimental studies is shown in Fig. 2a. Half-maximal closure time (hr) (Fig. 2b) represent the mean time to which wounds were 50 % closed among all five experiments. These data suggest that the wound repair response to ODE is dependent upon MyD88 signaling.

### Bone marrow chimeras demonstrate that MyD88 in lung resident cells is important for regulating ODE-induced AHR and diminished dynamic compliance

**Fig. 3:**
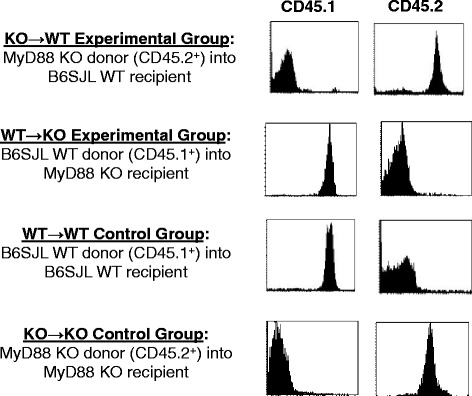
Validation of chimerism assured by cell surface expression of leukocyte CD45.1 versus CD45.2. Peripheral blood leukocytes were recovered from MyD88 bone chimeric mice at 6 weeks following irradiation and bone marrow reconstitution. CD45.1 (B6SJL origin) and CD45.2 (MyD88 KO origin) expression was evaluated by FACS. A representative histogram is shown

Our previous work demonstrated a critical role for MyD88-dependent pathways to acute ODE exposure *in vivo* because ODE-induced airway inflammation was nearly completely abrogated in MyD88 KO as compared to WT animals [4]. However, the importance of MyD88 in lung cell compartments is not known, and if evident, could lead to the future design of targeted cellular approaches. To understand the role of lung structural (predominately epithelial cell) versus hematopoietic-derived immune cells (including alveolar macrophages) to acute ODE treatment, we generated MyD88 chimeric mice as described in the Methods section. Discrimination between donor and recipient cells was determined by CD45.1 and CD45.2 expression on circulating leukocytes. Namely, the MyD88 WT B6.SJL congenic mice express the CD45.1 allele and the MyD88 KO mice express the CD45.2 allele. In pilot studies, the dose of ionizing radiation administered to recipient mice to ablate the bone marrow without inducing toxicity (death or lung pathology) was determined as 1000 rads (data not shown). At 8 weeks following bone marrow transfer, CD45.1 and CD45.2 expression on peripheral blood leukocytes was determined by FACS. A representative histogram of bone marrow chimeric mice is shown in Fig. 3. The two experimental groups of animals included the presence of MyD88 in lung structural cells, but not in hematopoietic cells (i.e. KO → WT) and vice versa (i.e. WT → KO). The other two groups (WT → WT and KO → KO) represented controls to rule out any non-specific effects of irradiation on measured responses. We could verify that the experimental chimeras and the WT → WT inflammatory profiles of irradiated saline and ODE treated control groups obtained in the present study were similar to those observed in non-irradiated MyD88 WT and KO mice as recently reported [4]. Any mice that demonstrated incomplete chimerism (i.e. greater than 10 % residual recipient phenotype) were excluded from the study. We show in Additional file 1: Figure S1 the lung histopathology of experimental chimeras treated with saline and ODE. Histopathology changes were slightly more pronounced in WT- > KO treated mice as compared to KO → WT animals.

**Fig. 4:**
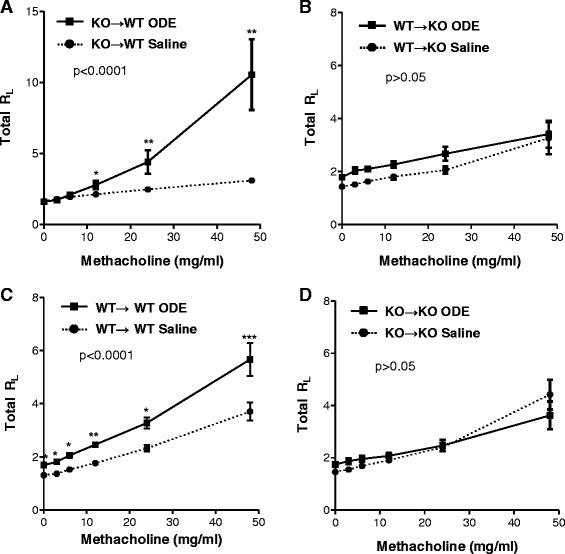
MyD88 in lung structural cells is important for regulating ODE-induced airway hyper-responsiveness (AHR). MyD88 bone marrow chimeric mice (donor → recipient) were treated with ODE (solid line) or saline (dotted line), and at three hr following *i.n.* inhalation treatment mice were tracheostomized, mechanically ventilated, and AHR to aerosolized methacholine (0, 3, 6, 12, 24, 48 mg) was measure and expressed as mean (±SEM) total lung resistance (R_L_). Chimeric groups depicted: **a** KO→WT; **b** WT→KO; **c** WT→WT; **d** KO→KO. Statistical difference between ODE and saline treatment groups was determined by two-way ANOVA (because there are 2 independent variables: treatment group and methacholine dose). If p-value less than 0.05, differences between saline and ODE at each methacholine doses were determined by *t*-test and asterisks denote significance (*p < 0.05, **p < 0.01, ***p < 0.001). *N* = 4–6 mice/group

Airway hyper-responsiveness (AHR) following large animal facility organic dust exposure is a central characteristic observed in humans [10, 21] and modeled in mice [10]. In general, the mechanisms governing AHR to organic dust exposures are not known, but we previously reported that ODE-induced AHR was abrogated in MyD88 KO mice [4]. In this study, we hypothesized that there would be equal contribution from lung structural cells and hematopoietic-derived immune cells in mediating MyD88-dependent AHR because neutrophil recruitment [22] and TNF-α [23] have been implicated in mediating non-allergic AHR. We utilized standard total lung resistance (R_L_) as the main AHR measurement because it reflects both narrowing of the conducting airways and parenchymal viscosity [24]. We found that ODE-induced AHR was MyD88-dependent only in lung structural cells (Fig. 4b). Namely, in the WT → KO experimental group, where MyD88 was absent in lung structural cells but not hematopoietic-derived immune cells, there was no evidence of ODE-induced AHR as compared to saline control (Fig. 4b). In contrast, in the KO → WT group, where lung structural cells expressed MyD88 and the hematopoietic-derived immune cells lacked MyD88, ODE-induced AHR remained intact (Fig. 4a). Importantly, control groups demonstrated similar responses to ODE as in our prior report, showing lack of ODE-induced AHR in MyD88 KO mice [4], which established that irradiation did not significantly alter baseline lung responsiveness.

**Fig. 5:**
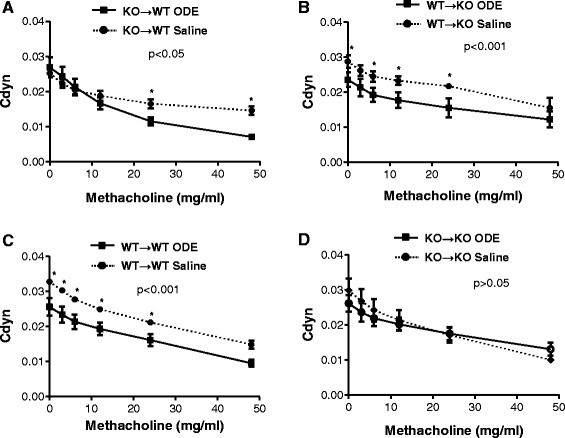
ODE-induced decreased dynamic compliance is predominately dependent on MyD88 in lung structural cells. MyD88 bone marrow chimeric mice (donor → recipient) were treated with ODE (solid line) or saline (dotted line), and at three hr following *i.n.* inhalation treatment, mice were tracheostomized, mechanically ventilated, and dynamic compliance (Cdyn) following aerosolized methacholine (0, 3, 6, 12, 24, 48 mg) was measured and expressed as mean (±SEM). Chimeric groups shown: **a** KO→WT; **b** WT→KO; **c** WT→WT; **d** KO→KO. Statistical difference between ODE and saline treatment groups was determined by two-way ANOVA (because there are 2 independent variables: treatment group and methacholine dose). If p-value less than 0.05, differences between saline and ODE at each methacholine doses were determined by *t*-test and asterisks denote significance (*p < 0.05). *N* = 4–6 mice/group

To broaden our understanding of how MyD88 affects lung function, we also investigated dynamic compliance (Cdyn), which reflects the elasticity of the lung parenchyma, but is also influenced by surface tension, smooth muscle contraction, and peripheral airway homogeneity [24]. First, in the WT → WT control group, ODE treatment decreased dynamic compliance as compared to saline (baseline measurement), and these differences remained with escalating concentrations of up to 24 mg methacholine (Fig. 5c). Similar to the WT → WT controls, the WT → KO experimental group where lung structural cells lack MyD88, displayed decreased dynamic compliance at baseline following ODE treatment (Fig. 5b), which was maintained with increasing doses of up to 24 mg methacholine (Fig. 5b). In comparison, in the KO → WT experimental group, where MyD88 was intact in lung resident cells, dynamic compliance differences between saline and ODE treatment were not observed until higher concentrations of methacholine were achieved (i.e. 24 mg and 48 mg, Fig. 5a). There was no difference in dynamic compliance between saline and ODE treatments in the KO → KO control group (Fig. 5d). Collectively, these pulmonary function studies in chimera mice reveal a critical role for MyD88-dependent signaling in lung radiation-resistant, tissue-resident cells for mediating ODE-induced AHR and decreased dynamic compliance.

### Important role of hematopoietic-derived immune cells for ODE-induced inflammatory cytokine/chemokine release

**Fig. 6:**
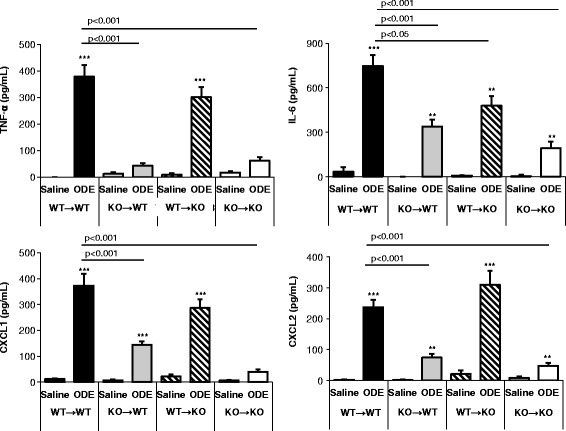
Important role of hematopoietic cells mediating ODE-induced inflammatory cytokine/chemokine release. MyD88 bone marrow chimeric mice (donor → recipient) were treated once with ODE or saline and at five hr post treatment, TNF-α, IL-6 and the murine neutrophil IL-8 chemokine homologs (CXCL1 and CXCL2) were determined in BALF. The data represent mean with standard error bars shown (*N* = 10–15 mice per ODE; *N* = 4–8 mice per saline treatment group). Statistical significance denoted by asterisks (*p* < 0.01, ***p < 0.001) as compared to respective saline treatment group. Line denotes statistical significance of chimera mice to WT mice treated with ODE

Inflammatory mediators, particularly TNF-α, have been suggested to mediate non-allergic AHR, and moreover, TNF-α, IL-6, and CXCL8/IL-8 (murine homologs: CXCL1 and CXCL2) production have been demonstrated in organic dust-induced airway disease in humans and mice [25]. Consistent with our previous work demonstrating a critical role for MyD88 in the release of these mediators [4], ODE-induced mediator release was significantly diminished, but not completely abrogated, in the KO → KO control group as compared to the WT → WT control group (Fig. 6). Compared to saline treatment, IL-6 and CXCL2 were increased in the ODE-treated KO → KO control group (Fig. 6). The current studies were expanded to identify the relative importance of MyD88 signaling in the lung parenchymal vs. hematopoietic compartments, where cytokine/chemokine production in the BAL fluid from control and experimental chimeric animals was determined. We demonstrate that ODE-induced TNF-α production is primarily dependent upon MyD88 signaling in hematopoietic cells, since minimal TNF-α production was observed in the KO → WT experimental group (Fig. 6). This was corroborated by the finding that TNF-α production was nearly fully restored following WT bone marrow cell reconstitution of MyD88 KO mice (WT → KO; Fig. 6). We also demonstrate that CXCL1 and CXCL2 production is strongly, but not completely, dependent upon MyD88-signaling in hematopoietic-derived cells (Fig. 6). ODE-induced IL-6 production was dependent upon both MyD88 signaling in lung resident and hematopoietic cells (Fig. 6). Thus, ODE-induced TNF-α, and to a lesser degree CXCL1 and CXCL2, release into the bronchoalveolar space is predominately dependent on MyD88 signaling from cells of bone marrow origin. In contrast, ODE-induced IL-6 production involves both MyD88-dependent lung radiation-resistant tissue resident and hematopoietic cells.

### Both hematopoietic and resident lung cells are important for MyD88-dependent neutrophil influx into the airways following ODE treatment

**Fig. 7:**
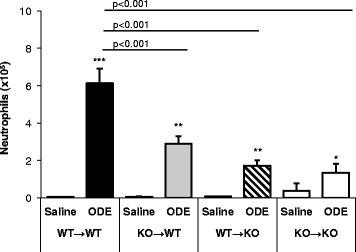
Lung structural and hematopoietic cells are required for ODE-induced neutrophil influx. MyD88 bone marrow chimeric mice (donor → recipient) were treated once with ODE or saline. At five hr post treatment, neutrophil recruitment into the bronchoalveolar lavage fluid was determined. The data represent mean with standard error bars shown of *N* = 10–15 mice per ODE and *N* = 4–8 mice per saline treatment groups. Statistical significance denoted by asterisks (*p < 0.05,**p < 0.01, ***p < 0.001) as compared to respective saline treatment group. Line denotes statistical significance of chimera mice to WT mice treated with ODE

Neutrophil influx is a hallmark of ODE-induced airway inflammatory responses; therefore, we next examined ODE-induced neutrophil influx in chimeric mice. As compared to the WT → WT control group, the experimental groups (KO → WT and WT → KO) and the KO → KO control group demonstrated a significant reduction in ODE-induced neutrophil recruitment (Fig. 7). Of note, the KO → KO saline group showed a slight increase in neutrophil influx compared to the other saline treatments; however, this was not statistically significant (Fig. 7). Nonetheless, results from the experimental chimera groups indicate that efficient neutrophil recruitment following ODE exposure depends on MyD88 signals mediated through both lung resident and myeloid cells.

## Discussion

Organic dusts from agriculture environments are complex mixtures containing a diverse array of microbial components and particulate matter that can rapidly activate airway inflammatory responses. Studies have shown partial reduction in organic dust-induced airway inflammation in TLR2-, TLR4-, and TLR9- deficient mice [4–6]. Moreover, IL-18R KO, but not IL-1R KO mice demonstrated slight reduction in airway inflammatory consequences to acute ODE treatment [4]. MyD88 is the common adaptor protein utilized by most TLRs (except TLR3) and IL-1R/IL-18R to transduce activation signals. We previously showed that AHR and inflammatory cell influx and mediator release into the bronchoalveolar space, but not lung parenchyma, following ODE challenge was nearly completely abrogated in MyD88-deficient animals [4]. We interpreted these findings to suggest a role for MyD88 in regulating epithelial cell inflammatory and repair consequences to complex organic dust exposures. However and in general, the role of MyD88 in epithelial cell functional was not well defined. Thus, our first objective of the current study was to define MyD88-dependent, epithelial cell responses following ODE exposures.

Mucociliary clearance and cellular migration/wound repair are important innate immune functions of epithelial cells that protect and repair the lung from deleterious effects of inhaled pollutants, allergens, and pathogens [26–28]. Exposure to cigarette smoke, bacterial pathogens, diesel exhaust particles, and organic dusts slow ciliary motility and decrease epithelial cell migration [14, 29–31]. Our studies demonstrate that MyD88 KO epithelial cells have normal baseline ciliary beat function and respond normally to the immunostimulatory non-TLR agent, procaterol (Fig. 1b). However, the ciliary motility slowing response to ODE treatment was blocked in MyD88 KO cells (Fig. 1a). The mechanisms to explain this response are likely due to the lack of a PKCε activation response in MyD88 KO cells. PKCε activation has been shown to mediate the ciliary slowing response to various environmental stimuli, including ODE [14]. Our previous data in MyD88 KO lung slices [4] and our current data in primary tracheal epithelial cells (Fig. 1c) support that ODE treatment slows CBF through PKCε activation downstream of MyD88 signaling. Next, our studies demonstrate that MyD88 signaling is necessary to recognize and respond to wounding of epithelial cells to microbial component-enriched environmental dusts, suggesting a fundamental role for MyD88 in lung injury and repair responses. Others have also shown that MyD88 is required for normal resolution of epithelial injury due to sclerosing agent-induced tracheal insult [32].

We next addressed whether MyD88 signaling in the structural lung cell compartment was responsible for regulating pulmonary function in response to organic dust. Humans and mice that are exposed once to ODE or organic dust environments demonstrate significant increases in AHR and/or decreased cross-shift pulmonary function [1, 10, 33–35]. AHR to organic dust exposures are not explained by TLR2 or TLR4 action alone [5, 6], but can be completely ablated in MyD88 KO animals [4]. We expanded our earlier observations of a MyD88-dependent AHR response to ODE to clearly demonstrate here that ODE-induced AHR is fully dependent upon MyD88 signaling in the lung resident cell compartment as opposed to hematopoietic-derived cells (Fig. 4). We did not anticipate this strong delineation in lung compartment effects because others have suggested that non-allergic AHR can be mediated by neutrophils or TNF-α [22, 23]. Our studies do not support a role for either neutrophils or TNF-α in ODE-induced AHR. This is because MyD88-driven TNF-α production was dependent upon hematopoietic-derived immune cells and neutrophil recruitment was influenced by both compartments. However, our findings are consistent with LPS-induced bronchoconstriction, where LPS-induced non-invasive AHR measurements were found to be strictly dependent on MyD88 signaling by radiation-resistant resident lung cells [36]. To our knowledge, the role of MyD88 signaling in airway smooth muscle and fibroblast function, independent of inflammatory cytokine production, has not been investigated, but might be important in the understanding of mechanisms and design of future therapies to target non-allergic mediated-AHR.

Asthma, hypersensitivity pneumonitis, and pulmonary fibrosis are associated with decreased lung dynamic compliance [37–40], and to varying degrees, these diseases have been reported in agriculture exposed humans [1, 41]. However, general knowledge of how MyD88 effects lung compliance has been largely unexplored. Using bone marrow chimera mice, we demonstrate that MyD88 signaling by radiation-resistant resident lung cells is predominately responsible for mediating decreased dynamic compliance in response to ODE. Similar to ODE-induced AHR, changes in dynamic compliance cannot be completely explained by ODE-induced inflammatory consequences. Interestingly, others have recently reported that silica-induced fibrosis is uncoupled from silica-induced inflammation in MyD88 KO mice [42]. Collectively, these findings highlight that MyD88-dependent signaling in lung resident cells (i.e. epithelial, endothelial, smooth muscle, myofibroblast cells) may represent potentially targetable lung cell populations to impact the adverse respiratory mechanics following complex, organic dust exposures.

Acute exposure to organic dust environments rapidly results in TNF-α, IL-6, and neutrophil chemoattractant (human CXCL8/IL-8 and murine CXCL1 and CXCL2) release. Our studies support that neutrophil influx and IL-6 production is dependent upon MyD88 signaling in both lung resident and hematopoietic-derived cells. TNF-α was almost fully dependent on MyD88-signaling in hematopoietic-derived immune cells. Release of the neutrophil chemoattractants (i.e. CXCL1 and CXCL2) was also predominately, but not entirely dependent on MyD88-signaling in hematopoietic immune cells. Although these studies were focused on delineating lung compartment-specific, MyD88-dependent functions, MyD88-independent responses also exist because ODE-induced IL-6 and CXCL2 release and neutrophil influx, albeit significantly dampened, in the KO → KO control group (Figs. 6 and 7). Next, future studies could investigate the role of non-cytokine mediators, such as surfactants and phospholipids, in mediating ODE-induced airway hyper-responsiveness.

In summary, our data demonstrate a key role for MyD88 in important epithelial cell functions to complex organic dust exposures *in vitro*, including modifying ciliary motility and wound repair. Furthermore, we established a strong delineation in lung cell compartment effects, with respiratory mechanics dependent upon MyD88-mediated signaling in lung resident cells, likely epithelial cells, which is suggested by our *in vitro* studies where MyD88 KO epithelial cells were refractory to ODE-induced dysfunction. Both lung resident and hematopoietic-derived (i.e. myeloid) cells were responsible for efficient neutrophil recruitment, and inflammatory cytokine/chemokine production appears to be driven mostly by contributions from peripheral immune cells. This work provides evidence that lung inflammatory mediators induced by organic dust exposures are not responsible for mediating respiratory dysfunction consequences. These findings might have implications in the design of future therapeutic approaches for agriculture workers with lung disease. Namely, we would propose that future investigations into emerging therapeutic approaches that target epithelial damage and lung remodeling (i.e. stem cells, epithelial-mesenchymal cells) are warranted as potential new directions to reduce complex organic dust and/or environmental toxin-induced lung disease.

## Additional file

Additional file 1: Figure S1. Lung parenchymal histopathology of experimental MyD88 bone marrow chimera (donor → recipient) mice treated once with saline or ODE. A representative murine lung section (hematoxylin and eosin stain, x10 magnification) is shown. Note that there is an absence of non-specific inflammation in saline-treated chimeric mice, and there is evidence of slight peribronchiolar and perivascular cellular cuffing following ODE treatment. Line scale is 100 μm. (PDF 699 kb)

## Abbreviations

AHR: Airway hyper-responsiveness
BALF: Bronchoalveolar lavage fluid
CBF: Ciliary beat frequency
COPD: Chronic obstructive pulmonary disease
CXCL1: Keratinocyte chemoattractant/KC
CXCL2: Macrophage inflammatory protein-2/MIP-2
ECIS: Electric cell substrate impedance sensing
FACS: Fluorescence activated cell sorting
IL: Interleukin
KO: Knock out
MyD88: Myeloid differentiation factor 88
ODE: Swine confinement facility organic dust extract
PBS: Phosphate buffered saline
PKC: Protein kinase C
TLR: Toll-like receptor
TNF-α: Tumor necrosis factor-alpha
WT: Wild-type
