# Supplementary figures and images for: MyD88 in lung resident cells governs airway inflammatory and pulmonary function responses to organic dust treatment
Source: Respir Res. 2015 Sep 16;16(1):111. doi: 10.1186/s12931-015-0272-9 (PMC4574163; doi:10.1186/s12931-015-0272-9)

**Saline Treatment**

**ODE Treatment**

**KO→WT**

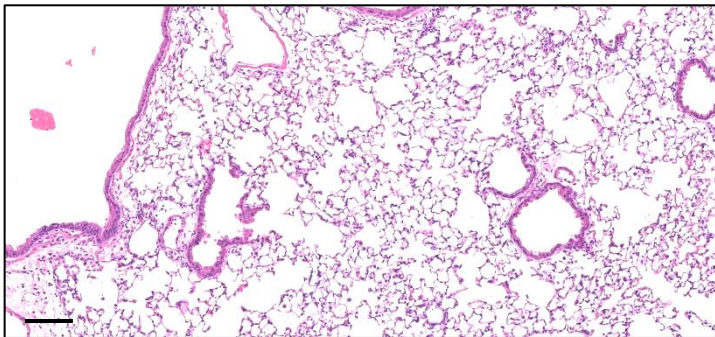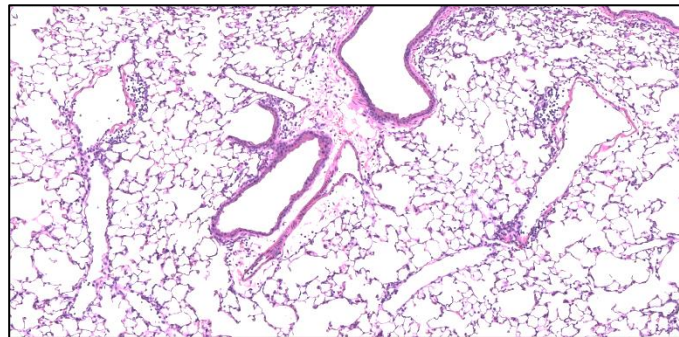

**WT→KO**

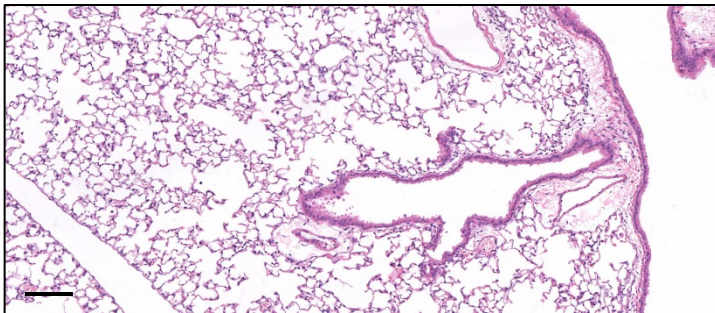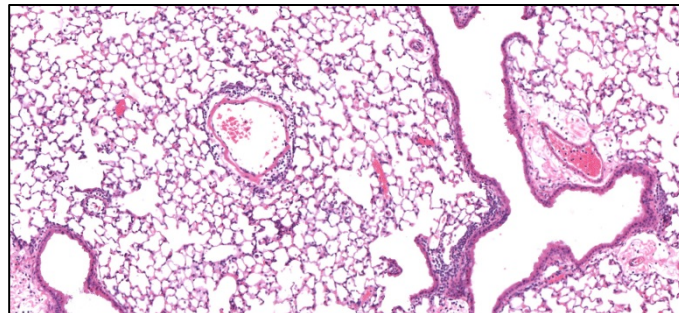

Supplement: Additional file 1: Figure S1. — Lung parenchymal histopathology of experimental MyD88 bone marrow chimera (donor → recipient) mice treated once with saline or ODE. A representative murine lung section (hematoxylin and eosin stain, x10 magnification) is shown. Note that there is an absence of non-specific inflammation in saline-treated chimeric mice, and there is evidence of slight peribronchiolar and perivascular cellular cuffing following ODE treatment. Line scale is 100 μm. (PDF 699 kb) [file 12931_2015_272_MOESM1_ESM.pdf]
